# Systemic Catecholaminergic Deficiency in Depressed Patients with and without Coronary Artery Disease

**DOI:** 10.3390/jcm10050986

**Published:** 2021-03-02

**Authors:** Uta Hoppmann, Harald Engler, Sabrina Krause, Edit Rottler, Julia Hoech, Franziska Szabo, Peter Radermacher, Christiane Waller

**Affiliations:** 1 Department of Neurology, Medical University of Berlin, 12203 Berlin, Germany; 2 Institute of Medical Psychology and Behavioral Immunobiology, University Hospital Essen, University of Duisburg-Essen, 45122 Essen, Germany; harald.engler@uk-essen.de; 3 Clinic of Psychosomatic Medicine and Psychotherapy, Medical University of Ulm, 89081 Ulm, Germany; sabrina.krause@uni-ulm.de (S.K.); edit.rottler@uniklinik-ulm.de (E.R.); julia.hoech@gmx.de (J.H.); franziska.szabo@gmx.de (F.S.); 4 Institute for Anaesthesiological Pathophysiology and Process Engineering, Medical University of Ulm, 89081 Ulm, Germany; peter.radermacher@uni-ulm.de; 5 Department of Psychosomatic Medicine and Psychotherapy, Paracelsus Medical University, Nuremberg General Hospital, 90419 Nuremberg, Germany

**Keywords:** depression, epinephrine, dopamine, coronary artery disease, inflammation, trier social stress test

## Abstract

Background: Stress and depression are known to contribute to coronary artery disease (CAD) with catecholamines (CA), altering the balance to a pro- and anti-inflammatory stetting and potentially playing a key role in the underlying pathophysiology. This study aimed to elucidate the impact of social stress on the CA system and inflammation markers in patients suffering from CAD and depression. Methods: 93 subjects were exposed to the Trier Social Stress Test (TSST). Based on the results of the depression subscale of the Hospital Anxiety and Depression Scale (HADS, German Version) and the presence/absence of CAD, they were divided into four groups. A total of 21 patients suffered from CAD and depression (+D+CAD), 26 suffered from CAD alone (−D+CAD), and 23 suffered from depression only (+D−CAD); another 23 subjects served as healthy controls (−D−CAD). Subjects were registered at 09:00 AM at the laboratory. A peripheral venous catheter was inserted, and after a 60-min-resting period, the TSST was applied. Prior to and 5, 15, 30, and 60 min after the stress test, plasma epinephrine, norepinephrine, and dopamine concentrations (High Performance Liquid Chromatography (HPLC)) were measured together with the inflammation markers interleukin-6 (IL-6) and monocyte chemotactic protein-1 (MCP-1). High-sensitive C-reactive protein (hs-CRP, Enzyme-linked Immunosorbent Assay (ELISA)) was measured prior to TSST. Results: (+D−CAD) and (+D+CAD) patients showed significantly lower epinephrine and dopamine levels compared to the (−D+CAD) and (−D−CAD) participants at baseline (prior to TSST). Over the whole measurement period after the TSST, no inter-group difference was detected. Partial correlation (controlling for age, gender and Body Mass Index (BMI)) revealed a significant direct relation between MCP-1 and norepinephrine (*r* = 0.47, *p* = 0.03) and MCP-1 and epinephrine (*r* = 0.46, *p* = 0.04) in patients with −D+CAD at rest. Conclusions: The stress response of the CA system was not affected by depression or CAD, whereas at baseline we detected a depression-related reduction of epinephrine and dopamine release independent of CAD comorbidity. Reduced norepinephrine and dopamine secretion in the central nervous system in depression, known as ‘CA-deficit hypothesis’, are targets of antidepressant drugs. Our results point towards a CA-deficit in the peripheral nervous system in line with CA-deficit of the central nervous system and CA exhaustion in depression. This might explain somatic symptoms such as constipation, stomach pain, diarrhoea, sweating, tremor, and the influence of depression on the outcome of somatic illness such as CAD.

## 1. Introduction

In patients with coronary heart disease (CAD), depression is associated with a 1.5 to 2.0-fold increased risk of mortality and morbidity [1]. Several genetic studies reveal pathways linking CAD and depression [2,3]. However, the underlying pathophysiology especially peripheral alteration of catecholamines (CA) and inflammatory system and reciprocal interaction, need further elucidation [4].

There is an on-going discussion on whether progression of arteriosclerosis is associated with inflammatory processes [5]. Studies on statins support the hypothesis that the development of arteriosclerosis is caused by inflammation. In fact, rosuvastatin lowers the level of C-reactive protein (CRP) and the incidence of cardiovascular events in healthy patient [6], and simvastatin has been shown to prevent accumulation of monocytes through the monocyte chemoattractant protein-1 (MCP-1) [7]. High levels MCP-1 are associated with negative effects on CAD like progression of arteriosclerosis through triggering inflammatory processes [8,9,10,11].

CA are possible candidates altering inflammatory processes [12]. Increased plasma levels of CA have been reported in clinical conditions such as congestive heart failure or myocardial infarction [13] with a CA-related change to pro-inflammatory and platelet-related pro-coagulatory responses, ultimately resulting in low-grade vascular hyper-inflammation [14,15]. Excessive CA responses could cause myocardial damage through inflammation. Genetic studies reveal a pathological link between inflammation and CA and an association to cardiovascular risk factors like BMI and hypertension [16]. A model for the development of CAD suggests that both inflammation and changes in the CA system are essential [17]. An investigation on men with carotid plaques support the link between dysfunction in the CA system in association with inflammation [18]. Acute stress might even trigger the rupture of arteriosclerotic plaques through the imbalance of the CA system [19]. In summary, both inflammation and CA play an important role in the development and progression of CAD, and there is evidence that high levels of the CA are related to a pro-inflammatory state.

Genetic studies and experimental data linking cardiovascular diseases and depression and neuroimmunology might play a key role in the underlying pathology [20]. Data supporting the role of inflammation in depression are extensive: depression is associated with increased inflammatory biomarkers like elevated CRP and Interleukin 6 (IL-6) [21,22,23], and a pro-inflammatory state might worsen the outcome of depression [24]. A recent metanalysis characterized an inflammation profile in depression with elevated IL-6, tumor necrosis factor (TNF)-alpha, other cytokines, and chemokines [21]. In line with these data, antidepressant treatment significantly decreased TNF-alpha and IL-6 [25,26,27]. 

Changes in the homeostasis of the sympathetic nervous system characterized by sympathetic overactivity and/or vagal suppression is well documented in depression [20,28,29]. A recent study discussed a parasympathetic predominance as a risk factor for developing depression [28]. Results on norepinephrine levels are conflicting, since unchanged [30] and increased plasma norepinephrine levels were reported [31]. Likewise, both reduced [30] and increased urinary norepinephrine excretion have been observed [31]. 

Only a few studies, however, have been dedicated to the examination of the CA system, in order to elucidate on a possible pathophysiological link between depression and CAD. Elevation in the heart rate (HR) and a reduction in HR variability have been widely shown in depressed patients, both with and without CAD [32,33] and linked with cardiac mortality after myocardial infarction [34]. 

A review by Benarroch gives a new insight to a sympathetic-cholinergic anti-inflammatory pathway with suppression of anti-inflammatory cytokines [35]. Therefore, any imbalance in the CA system with a shift to a pro-inflammatory setting might be a potential pathophysiological link to understand the higher mortality and morbidity in patients with CAD and comorbid depression [36], indicating that both disturbed adrenergic neurotransmitter pathways and chronic inflammation conditions have an influence on the genesis and progression of the arteriosclerosis [20,21,37]. 

Therefore, the present study aimed to clarify the association between depression and CAD, inflammation and stress in the CA system. The first aim was to detect an altered stress response in patients with depression. For this purpose, we used a social stressor, the Trier Social Stress Test (TSST), as there is substantial evidence that social stress may be even more significant than e.g., physical stress [27]. A second aim was to elucidate the association between inflammation and the CA system, which might alter the balance of inflammation markers. Therefore, we investigated the baseline and acute social stress-induced activation of the adrenomedullary system and dopamine autocrine/paracrine system in a CAD patient group with (+D+CAD) and without depressive symptoms (−D+CAD), and compared them to both patients suffering from depressive symptoms without CAD (+D−CAD) and healthy controls (−D−CAD). To evaluate the effect of the CA system on the pro-inflammatory state, we examined plasma IL-6, hsCRP, and MCP-1 at baseline and during as well as after the acute social stress-induced activation. We hypothesized that (i) the peripheral CA-system is altered in patients suffering from depression and, (ii) the alteration of the peripheral CA-system is linked to a pro-inflammatory state. 

## 2. Material and Methods

### 2.1. Participants

The study was approved by the local Ethics Committees of the Faculty of Medicine, University of Ulm and Medical University of Hanover, Germany, and was conducted according to the Declaration of Helsinki. After full description of the study, all study participants signed informed consent before the TSST and registration at the laboratory. 

Ninety-three subjects participated in the study. Based on the results of the depression subscale of the Hospital Anxiety and Depression Scale (HADS, German Version) and the diagnosis CAD, they were divided into four groups: Forty-seven patients suffered from CAD and were recruited from the cardiovascular wards. Recent coronary angiograms confirmed the presence of CAD and were performed within 6 months prior to the study. Twenty-three depressed patients without CAD were recruited in the psychiatric or psychosomatic departments of the University of Ulm. Twenty-three healthy subjects were recruited by advertising in newspapers and flyers and were individually matched for gender and age before participating in the study. Medical diagnoses of all patients were made based on clinical observation and medical records. Quantities of cardiac and antidepressant medications were recorded, and Canadian Cardiovascular Society (CCS) and New York Heart Association functional (NYHA) classifications were determined. Exclusion criteria included significant major medical illnesses likely to confound acute stress hormone levels, pregnancy, other Axis I psychiatric diagnoses as well as current psychotic symptoms or drug/alcohol abuse. Female subject parameters were taken either at days 1–3 of regular menstrual cycles or after menopause. Hormonal contraceptives or treatments were excluded. 

### 2.2. Procedure

Subjects reported to the laboratory between 8:00 and 9:00 a.m. Height and weight were recorded, and body mass index (BMI) was calculated according to standard formula. We applied the standard protocol of the widely used TSST [28] which reliably induces a twofold to threefold neuroendocrine and cardiovascular response. The TSST combines a social and cognitive stressor comprising a 5-min anticipatory stress following a short introduction and 5-min public speech (simulated job interview) and five minutes of mental arithmetic calculation, both done in front of an audience. After the TSST, subjects were asked to remain seated in a quiet room for another 60 min to recover. 

For assessment of circulating neuroendocrine markers, a peripheral venous catheter was inserted immediately after registration followed by a 60 min resting period to avoid changes in neuroendocrine markers due to the pain of puncturing. Immediately before and after the stress test as well as 5, 15, 30, and 60 min after the whole procedure, blood was drawn into EDTA-coated monovettes (ethylenediaminetetraacetic acid; Sarstedt, Nuembrecht, Germany) to determine total plasma epinephrine, norepinephrine, and dopamine levels. Blood pressure (BP) and heart rate were recorded seated at the blood sampling time points (boso-medicus control, Jungingen, Boso, Germany). Mean arterial blood pressure (MAP) was calculated using the standard formula. 

### 2.3. Blood Sampling and Biochemical Analyses

Monovettes were centrifugated at 3000 revolutions per minute (rpm) at +4 °C for 10 min, and the plasma was stored at −80 °C for further analysis. Epinephrine, norepinephrine and dopamine concentrations were measured by high-pressure liquid chromatography (HPLC; Chromsystems, Gräfelfing, München, Germany). The detection limits for all parameters were <15 ng/L. Within-assay coefficients of variance (CV) were 7.9% for epinephrine, 3.9% for norepinephrine, and 11.4% for dopamine. Between-assay CVs were 6.5% for epinephrine, 5.9% for norepinephrine, and 12.7% for dopamine. Plasma concentrations of MCP-1, IL-6 and hs-CRP were measured by enzyme-linked immunosorbent assay (ELISA) (MCP-1 and IL-6: Human Quantikine ELISA, R&D Systems, Minneapolis, MN, USA; hs-CRP: IBL International, Hamburg, Germany) according to the manufacturer’s protocols. The sensitivity of the assays was 10 pg/mL for MCP-1, 0.70 pg/mL for IL-6, and 0.02 µg/mL for hs-CRP. Mean inter- and intra-assay CVs were <8%.

Plasma concentrations of MCP-1, IL-6, epinephrine, norepinephrine and dopamine were quantified prior to and 5, 15, 30, and 60 min after the stress test. Since hs-CRP is not influenced by stress, it was only measured prior to TSST. We focused especially on inflammatory markers which might be linked to catecholamines, depression and CAD and might reflect a pro-inflammatory state, i.e., hs-CRP as a general marker for inflammation, IL-6 as cytokine, and MCP-1 as chemokine.

Due to technical issues we had missing data for IL-6 and MCP-1 (+D+CAD: IL-6: *n* = 19, MCP-1: *n* = 21, hs-CRP: *n* = 20, −D+CAD: IL-6: *n* = 21, MCP-1: *n* = 25, hs-CRP: *n* = 25, +D−CAD: IL-6: *n* = 20, MCP-1: *n* = 22, hs-CRP: *n* = 22, −D−CAD: IL-6: *n* = 12, MCP-1: *n* = 13, hs-CRP: *n* = 13). There was one missing data for epinephrine, norepinephrine, and dopamine in −D−CADand −D+CAD.

### 2.4. Psychometry

#### 2.4.1. Assessment of Depression

The German version of the 7-item hospital anxiety and depression scale (HADS) was used to rate symptom levels of depression, giving rise to a total depressive symptom score between 0 and 21. HADS values were 8–10 for mild, 11–14 for moderate, and 15–21 for severe depressive moods. Results of the HADS symptom scores are summarized in Table 1. The Structured Clinical Interview for DSM-IV (SCID-IV) to evaluate the diagnosis of depression was carried out with all participants. Thirty-two patients met the DSM-IV criteria for major depression, 7 patients for major depression in the last few months in partial remission, and 5 patients for dysthymia. Seven patients also met DSM-IV criteria for an anxiety disorder, 2 for panic disorder, and 1 for anxiety not otherwise specified (NOS). 

#### 2.4.2. Assessment of Anxiety, Stress and Physical Symptoms

Psychological stress is associated with anxiety and induces physical symptoms. Depression and anxiety are closely linked. In order to test the effectivity of induced psychological stress and to control for possible confounding variables, we used the following questionnaire: (1) State-Trait Anxiety Inventory (STAI) was developed by Spielberg (German version translated by Laux, [38]). We only used the state form of the questionnaire. Patients rated their actual level of anxiety on a four-digit scale and the questionnaire consists of 20 items. (2) Primary Appraisal Secondary Appraisal (PASA) is based on the appraisal theory of Lazarus and focused on cognitive evaluation of the situation. PASA was developed by Gaab and consists of 16 items with a sixth-digit scale [39]. (3) Complaint-list (Beschwerdenliste (BL) Hogrefe Testzentrale: Herbert-Quandt-Str. 4, 37081 Göttingen, Germany) was developed by Zerssen 1976 and consists of 24 items. The Complaint-list is assessing physical symptoms like Fatigue, weakness, pyrosis, et cetera on a four-digit scale [40]. 

### 2.5. Statistical Analyses

Data were analysed using SPSS statistical software package version 25.0 (SPSS Inc., Chicago, IL, USA). Testing was two-tailed with the significance level set at *p* < 0.05. All results are shown as mean ± standard deviation (SD). CCS and NYHA data are given as means ± interquartile range (IQR). Normal distribution of data was tested by nonparametric Kolmogorov-Smirnov test. Greenhouse-Geisser correction for repeated measures was applied. Since CA markers and HADS depression score were normally distributed, analyses were realized as follows: Univariate analyses of variance were calculated across the four subject groups (+D+CAD, +D−CAD, −D+CAD, −D−CAD) with group as independent and baseline CA as dependent variables. Differences in CA stress reactivity between the four groups were calculated by ANCOVAs for repeated measures with group being the independent variable with four characteristics (4 groups) and the CA markers (i.e., epinephrine, norepinephrine, and dopamine) being repeated dependent variables (5 repetitions). To prevent overcontrolling given our sample size, we first controlled for age, gender and beta-blockers as three à priori defined covariates known to significantly influence stress reactivity. Secondly, BMI, antidepressants, ACE inhibitors, and AT1-antagonists were tested separately in order to eliminate potential medication-related effects. Since inflammatory markers were not normally distributed, we calculated partial correlation with age, gender, and BMI as three à priori defined confounding variable to explore the association between inflammation markers and CA system. We separately controlled for medication (antidepressants, ACE inhibitors, and AT1-antagonists, statins).

## 3. Results

### 3.1. Subjects’ Characteristics

Table 2 presents the biological characteristics of the four groups. BMI of (+D+CAD) patients was significantly higher when compared to (–D+CAD) and (−D−CAD) (*p* < 0.01) and (+D−CAD) patients (*p* < 0.05). (−D+CAD) patients were significantly older (*p* < 0.01) than the other study groups. MAP and HR showed comparable stress-induced increases in all groups. The CCS classification was 1.5 ± 1 in (+D+CAD) and 1.2 ± 0 in (−D+CAD) patients. NYHA classification was 1.4 ± 1 in both CAD groups. 

Table 1 summarizes the psychological data of the four groups. The highest score in BL was found in the (+D−CAD) group with significantly higher values compared to the (−D+CAD) and (−D−CAD) groups (*p* < 0.000). STAI S1 showed the highest scores in the (+D−CAD) group compared to the other three groups (*p* < 0.000) followed by the (+D+CAD) group compared to the (−D+CAD) and the (−D−CAD) groups (*p* = 0.029 and *p* < 0.005, respectively). Again, STAI S2 values were the highest in the (+D−CAD) group compared to the (−D+CAD) and the (−D−CAD) groups (*p* < 0.000). STAI S2 values of the (+D+CAD) group were significantly higher compared to the (−D−CAD) group (*p* < 0.000). Significant differences between the groups were found in the PASA stress index with again the highest values in the (+D−CAD) group compared to the (−D+CAD) and the (−D−CAD) groups (*p* = 0.009 and *p* = 0.003), respectively.

Table 3 summarizes the drugs of the four groups. In the (+D−CAD) group, 61% reported to take antidepressants and 4% to take beta-blockers. The (+D+CAD) group reported to take antidepressants (24%), beta-blockers (71%), angiotensinconverting-enzyme-(ACE)-inhibitors (81%), and Angiotensin II receptor type-(AT1)-antagonists (14%). The (−D+CAD) group reported to take beta-blockers (86%), ACE-inhibitors (73%), and AT1-antagonists (23%). The (−D−CAD) group reported to have no drug treatment.

### 3.2. SAM-Axis at Baseline and during Stress Response

**Figure 1:**
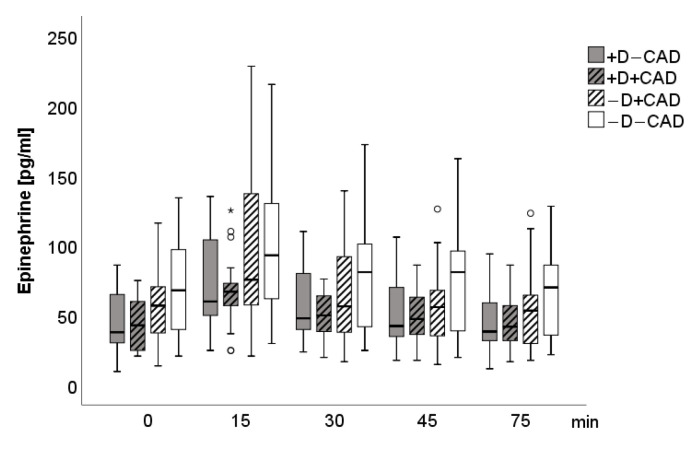
Boxplot of epinephrine for each measurement time. Circle: ordinary outliner defined as one and a half boxlength, asterisk: extreme outliner defined as above one and a half to threefold boxlength.

**Figure 2:**
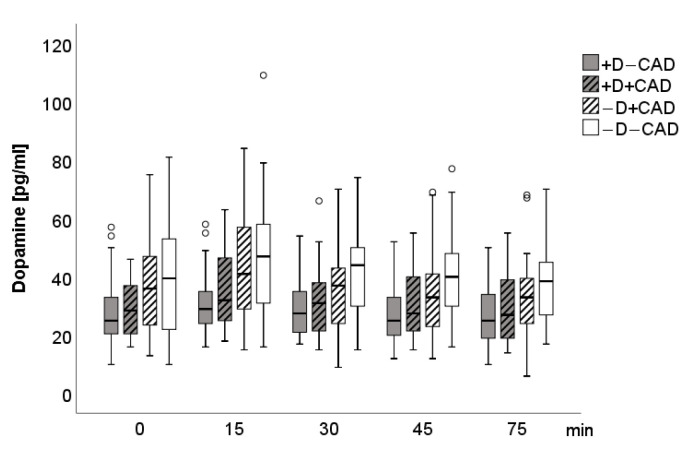
Boxplot of dopamine for each measurement time. Circle: ordinary outliner defined as one and a half boxlength.

**Figure 3:**
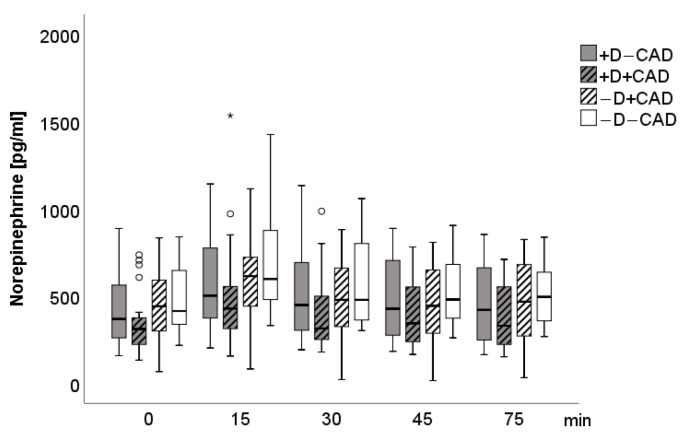
Boxplot of norepinephrine for each measurement time. Circle: ordinary outliner defined as one and a half box-length, asterisk: extreme outliner defined as above one and a half to threefold box-length.

A significant main group effect (F(3; 83) = 3.63, *p* = 0.016) was found for epinephrine when controlling for age, gender, and beta-blockers, including the four groups. Epinephrine levels were significantly lower in the (+D−CAD) (F(1; 39) = 5.73, *p* = 0.022) and the (+D+CAD) groups (F(1; 38) = 4.63, *p* = 0.038) when compared to the (−D−CAD) group. Neither significant main time (F(1.9; 160.1) = 1.07, *p* = 0.342) nor main group-by-stress interaction effects (F(5.8; 160.1) = 1.13, *p* = 0.349) were found for epinephrine. Additional adjustments for cardiovascular and antidepressant drugs as covariates did not significantly affect the results. For dopamine levels, there was a significant time (F(2.4; 201.6) = 4.94, *p* = 0.005) as well as a group effect (F(3; 83) = 3.62, *p* = 0.016) with significantly lower dopamine levels in the (+D−CAD) when compared to the (−D−CAD) group (F(1, 39) = 9.50, *p* = 0.004) and a trend to lower dopamine levels compared to the (−D+CAD) group (F(1; 42) = 3.35, *p* = 0.075). No significant group-by-stress interaction effect for dopamine was found (F(7.3; 201.6) = 1.70, *p* = 0.108). Again, no significant changes in the results were seen after additional adjustments for cardiovascular and antidepressant drugs as covariates. Norepinephrine values showed a time effect (F(2.4; 198.5) = 4.27, *p* = 0.011); however, no group (F(3; 83) = 1.68, *p* = 0.178) or group-by-stress effects (F(7.2, 198.5) = 1.05, *p* = 0.398) were detectable between the four groups. Figure 1, Figure 2 and Figure 3 describe the time course of SAM-Axis.

### 3.3. SAM-Axis and Inflammatory Markers

**Figure 4:**
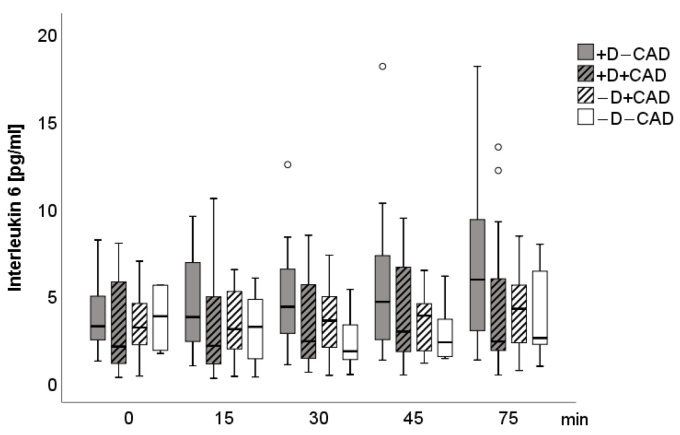
Boxplot of Interleukin 6 for each measurement time. Circle: ordinary outliner defined as one and a half boxlength.

**Figure 5:**
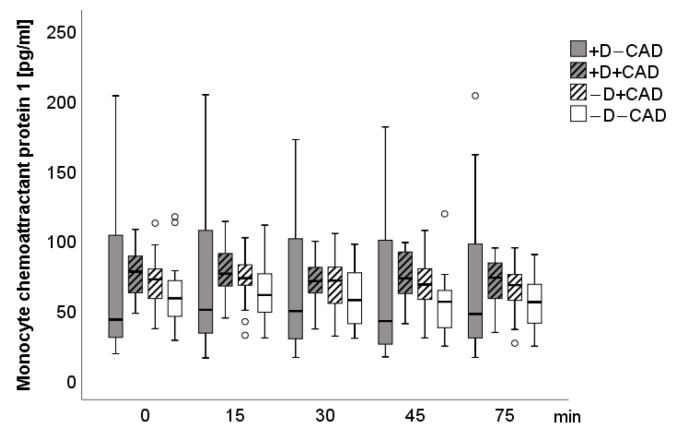
Boxplot of Monocyte chemoattractant protein 1 for each measurement time. Circle: ordinary outliner defined as one and a half boxlength.

Figure 4 and Figure 5 describe the time course of IL-6 and MCP-1. At baseline, significant partial correlations were found between MCP-1 and norepinephrine (*r* = 0.47, *p* = 0.03), MCP-1 and epinephrine (*r* = 0.46, *p* = 0.04), IL-6, and dopamine (*r* = 0.54, *p* = 0.04) in patients with −D+CAD. Five minutes after stress, a significant partial correlation between norepinephrine and MCP-1 (*r* = 0.44, *p* = 0.05) in patients with −D+CAD was visible. No other significant group effects, interaction effects, or correlations were detected. After additional adjustment for medication, significant correlations only remained between MCP-1 and norepinephrine and between MCP-1 and epinephrine prior to TSST (at baseline). In particular, when adjusting for statins and AT1-antagonists, the partial correlations between norepinephrine and MCP-1 and between IL-6 and dopamine no longer showed significance. No group effect was found at baseline for hs-CRP (+D+CAD: 3.2 mg/L ± SD: 4.9, −D+CAD: 1.4 mg/L, ±SD: 1.1, +D−CAD: 2.1 mg/L, ± SD: 2.8, −D−CAD: 2.0 mg/L, ± SD: 3.5).

## 4. Discussion

In our study, we used a well-established social stress test, which enabled us to study baseline and stress-induced stress-hormone changes with a high level of consistency [41]. Using this method, we were able to compare epinephrine und dopamine plasma levels in the four patient groups, in order to study both the influences of depression and CAD on the peripheral CA system and inflammation markers. The main results were that: (i) epinephrine and dopamine plasma levels were lower at baseline in the depressed patients regardless of CAD status ((+D−CAD) and (+D+CAD)) when compared to the non-depressed groups ((−D+CAD) and (−D−CAD)), whereas norepinephrine levels were not affected; and (ii) during acute social stress, CA responses were maintained in all groups, which indicates a preserved ‘fight or flight’ response to social stress.

Our results suggest a peripheral deficit in the adrenergic hormonal and dopamine autocrine/paracrine systems due to depression. Interestingly, albeit CA concentrations were lower at baseline in the depressed patient groups, social stress responses were maintained in the peripheral CA system in all groups. This is consistent with numerous studies that show that the CA-related ‘fight-or-flight’ response is highly preserved [42,43,44]. Only few studies focused on peripheral CA levels in relation to depression. Human studies on the relationship between plasma epinephrine and dopamine and depression are rare, and results show high inconsistencies due to complex in vivo processes of CA release and degradation [30,31,45,46]. Depression is sometimes described as being related to a peripheral hyper-responsiveness to norepinephrine [47], and increased norepinephrine plasma levels [48]. The latter result is in contrast to our finding of unchanged baseline norepinephrine levels. However, these authors investigated younger subjects (mean age 36.9 vs. 50 and 56 in our +D−CAD and +D+CAD groups, respectively) without any continuous medication [47]. Since all CAD patients have to take medication, we could not study CAD patients without medication. We controlled for putative medication effects, and in all CAD patients, the stress reactivity was preserved. Hence, our results might not relate to young, but otherwise cardiovascular healthy patients with depression. Beta-blockers in particular influence the norepinephrine levels [49]. Since they are frequently used in CAD patients due to well-established positive effects on long-term outcome [50], this indicates an important role of medication on norepinephrine levels, and thus may also explain the conflicting results. Recently, animal studies revealed impaired adrenal medullary function with lower epinephrine plasma levels in a model of depression due to chronic stress [51]. In addition, Lechin et al. [52] described maximum neural sympathetic activity as well as sympathetic hypoactivity of the adrenal glands in patients with major depression when compared to healthy controls. Hence our results could point towards CA exhaustion.

Dopamine is one of the main monoamine neurotransmitters in the brain and plays an important role in the pathophysiology of depressive disorders. However, the sources and pathophysiological role of dopamine outside the brain are not fully investigated. One possible source of plasma dopamine are sympathetic noradrenergic nerves [53], so the result of lower dopamine level would suggest a CA exhaustion as well.

Several lines of evidence suggest that dopamine may act as a regulator of local organ function [54]. Peripheral dopamine plasma values are rarely reported in scientific literature, despite the fact that the dopaminergic system plays an important role in the cardiovascular system [44]. Dopamine might play a role in hypertension [55] and the role of a dopaminergic renal system in hypertension is discussed [56]. A recent study showed an inverse correlation between plasma dopamine and C-peptide concentrations in patients with diabetes, emphasizing modulating effects [57]. Therefore, a dopamine imbalance may point towards insufficient controlled risk factors in patients with depression and CAD. In patients with depression only, it might indicate the risk for hypertension and diabetes.

Immunosuppressive effects of catecholamines have been demonstrated both in vitro and in vivo during endotoxemia in human volunteers [12,58,59]. We could not demonstrate a link between lower epinephrine and dopamine levels at baseline or stress. This might be due to antidepressant medication, which per se could alter inflammation markers [25,26,27]. Antidepressant medication could decrease peripheral markers like IL-6 [60]. Therefore, missing the link between CA and inflammation in depression in our study might be due to antidepressant medication. A pro-inflammatory state is more common in atypical depression compared to melancholic depression [21]. We did not distinguish between atypical and melancholic depression and missed a chance to detect a pro-inflammatory state. Furthermore, missing the link between CA and inflammation could have occurred to the assay method we used for the inflammatory markers.

We could show a significant positive correlation between epinephrine, norepinephrine and MCP-1, which plays a major role for the development of arteriosclerosis [61], in patients with CAD. At rest, norepinephrine and epinephrine levels were directly related to MCP-1 concentrations in patients with CAD, indicating a link between the CA and inflammation. Only few studies focused on a possible association between epinephrine and MCP-1, and could establish a direct correlation, which is in accordance with our study [62,63]. In CAD patients, MCP-1 is mainly associated with negative effects like progression of inflammatory arteriosclerosis by activation of macrophages and monocytes, and high levels of MCP-1 are both associated with risk of CAD and inflammation [8,9,10,11]. In addition, beta-blockers influence the level of inflammation markers like IL-6, MCP-1, TNF-α, and TNF-β1 through blocking interleukin-1β [64]. Statins might prevent excessive monocyte recruitment through MCP-1 [7]. Finally, AT2-antagonists have been referred to for MCP-1 as well [65]. We showed that the association between norepinephrine and MCP-1 after the TSST was affected by medication, pointing towards a disease-modulating aspect of these drugs. The importance of modifying the norepinephrine response by drugs is demonstrated by the discussion of adrenergic escape being a risk factor for therapeutic failure in chronic heart failure [49].

We could demonstrate that the peripheral CA-system is altered in patients suffering from depression, but we missed the chance to link this alteration to a pro-inflammatory state in depression, especially in patients with CAD and depression. This might be due to the disease modifying aspects of the drugs that these patients took. In this study, we could only focus on some candidates of the inflammatory system, which might be linked to catecholamines, depression and CAD, i.e., IL-6 and MCP-1. However, there are other cytokine (e.g., interleukin-1, tumor necrosis factor-alpha) [66,67] which might better reflect allostatic changes in depression and CAD.

The main limitation of this study is a low sample size, however, other experimental studies using the TSST have similar sample sizes [68,69,70,71,72,73]. Since more male patients suffered from CAD, we could only include a few female subjects. These results apply primarily to male patients. There was a significant age and BMI difference between the groups. In particular, BMI might modulate the effects of an inflammatory system.

## 5. Conclusions

The main results that we found are reduced circulating epinephrine and dopamine levels in depression, regardless of the CAD status. This might reflect CA exhaustion. In patients with CAD and without depression, epinephrine might play a role in inflammation, especially through MCP-1, and drugs might have a disease-modulating effect through epinephrine. We could not confirm an association of CA system and inflammation in depression with and without stress.

Further studies should focus on peripheral change in the CA system in depression. This might elucidate the pathophysiology of bodily symptoms in depression. Studies should focus on the mutual effect of CA system and inflammation, as such an interaction could explain the negative effects of depression on CAD. In this context, the effects of antidepressant drugs should be investigated further.

## Figures and Tables

**Table 1:** Psychological data.

	+D−CAD (*n* = 23)	+D+CAD (*n* = 21)	−D+CAD (*n* = 26)	−D−CAD (*n* = 23)
BL	32.3 ± 10.1 (17–55)	27.9 ± 11.6 (3–50)	14.4 ± 10.7 (1–37)	9.5 ± 7.7 (0–24)
HADS anxiety	12.5 ± 3.3 (3–18)	10.7 ± 4.2 (3–18)	5.7 ± 3.5 (0–12)	3.7 ± 2.6 (0–9)
HADS depression	11.9 ± 3.9 (8–18)	11.1 ± 2.4 (8–15)	3.6 ± 2.4 (0–7)	2.0 ± 1.9 (0–7)
PASA threat	3.6 ± 1.3 (1.3–5.8)	3.4 ± 1.2 (1.0–6.0)	2.7 ± 1.0 (1.0–5.0)	2.5 ± 1.0 (1.0–4.3)
PASA challenge	4.4 ± 0.7 (3.0–5.3)	3.9 ± 1.0 (1.8–6.0)	3.9 ± 0.8 (2.8–5.5)	3.8 ± 0.7 (2.3–5.0)
PASA primary appraisal	4.0 ± 0.9 (2.4–5.4)	3.7 ± 1.0 (1.4–5.8)	3.3 ± 0.8 (2.3–4.8)	3.2 ± 0.7 (2.0–4.4)
PASA self-concept	3.5 ± 1.4 (1.0–5.5)	4.1 ± 1.0 (2.5–5.8)	4.1 ± 1.3 (1.0–6.0)	4.4 ± 1.2 (1.8–6.0)
PASA control expectancy	4.4 ± 0.9 (3.0–6.0)	4.5 ± 0.7 (3.0–6.0)	5.1 ± 0.8 (3.0–6.0)	4.9 ± 0.9 (1.8–6.0)
PASA secondary appraisal	4.0 ± 0.9 (2.4–5.3)	4.3 ± 0.7 (3.4–5.4)	4.6 ± 0.9 (2.9–6.0)	4.7 ± 0.8 (2.9–5.8)
PASA stressindex	0.02 ± 1.7 (−2.6–2.8)	−0.7 ± 1.4 (−4.0–1.1)	−1.4 ± 1.4 (−3.8–1.4)	−1.5 ± 1.3 (−3.8–0.9)
STAI S1	53.6 ± 10.4 (32–73)	40.6 ± 9.9 (29–64)	32.6 ± 9.6 (20–58)	30.7 ± 7.7 (21–44)
STAI S2	56 ± 11.9 (34–77)	47.3 ± 9.8 (27–66)	39.6 ± 13.1 (22–66)	31.9 ± 9.3 (21–58)

BL: Beschwerdenliste; HADS: Hospital Anxiety and Depression Scale; PASA: Primary Appraisal Secondary Appraisal; STAI: State-Trait Anxiety Inventory.

**Table 2:** Biological characteristics.

	+D−CAD (*n* = 23)	+D+CAD (*n* = 21)	−D+CAD (*n* = 26)	−D−CAD (*n* = 23)
Age (y)	50 ± 7.3 (39–69)	56 ± 8.6 (41–72)	63 ± 9.6 * (44–75)	52 ± 8.4 (41–71)
Gender (m/f)	18/5	18/3	21/5	20/3
BMI (kg/m^2^)	27.3 ± 3.2 (19.3–32.7)	29.7 ± 3.5 (21.3–36.7) ^#^	26.8 ± 3 (19.9–32.1)	26.4 ± 2.9 (21.0–33.1)
CCS		1.5 ± 1	1.2 ± 0	
NYHA		1.4 ± 1	1.4 ± 1	

* *p* ≤ 0.05 vs. the other three groups, ^#^ *p* < 0.05 vs. (−D−CAD) and (−D+CAD) groups. Values are given as means ± SD (range), for CCS and NYHA data are given as means ± IQR. m, male; f, female; BMI, body mass index; CCS, Canadian Cardiovascular Society grading; NYHA, New York Heart Association functional classification.

**Table 3:** Summary of the drugs.

	+D−CAD (*N* = 23, *n*/%)	+D+CAD (*N* = 21, *n*/%)	−D+CAD (*N* = 26, *n*/%)	−D−CAD (*N* = 23, *n*/%)
β blockers	1 (4)	15 (71)	23 (86)	0 (0)
ACE-inhibitors	0 (0)	17 (81)	19 (73)	0 (0)
AT1-antagonists	0 (0)	3 (14)	6 (23)	0 (0)
Ca-antagonists	0 (0)	4 (19)	5 (19)	0 (0)
diuretics	0 (0)	13 (62)	9 (35)	0 (0)
Statins	1 (4)	19 (91)	24 (92)	0 (0)
Antidepressants	14 (61)	5 (24)	0 (0)	0 (0)

N & n, number; ACE: angiotensinconverting-enzyme; AT1: Angiotensin II receptor type 1; β: beta; Ca: calcium channel.

## Data Availability

The data presented in this study are available on request from the corresponding author. The data are not publicly available due to privacy. We did not inform the participants about publicly accessible data.

## References

[1] Leung Y.W., Flora D.B., Gravely S., Irvine J., Carney R.M., Grace S.L. (2012). The Impact of Premorbid and Postmorbid Depression Onset on Mortality and Cardiac Morbidity among Patients with Coronary Heart Disease: Meta-Analysis. Psychosom. Med..

[2] Amare A.T., Schubert K.O., Tekola-Ayele F., Hsu Y.-H., Sangkuhl K., Jenkins G., Whaley R.M., Barman P., Batzler A., Altman R.B. (1996). The association of obesity and coronary artery disease genes with response to SSRIs treatment in major depression. J. Neural. Transm..

[3] Akosile W., Voisey J., Lawford B., ColquhounC D., Young R.M., Mehta D. (2018). The inflammasome NLRP12 is associated with both depression and coronary artery disease in Vietnam veterans. Psychiatry Res..

[4] Huffman J.C., Celano C.M., Beach S.R., Motiwala S.R., Januzzi J.L. (2013). Depression and Cardiac Disease: Epidemiology, Mechanisms, and Diagnosis. Cardiovasc. Psychiatry Neurol..

[5] Lichtman A.H., Binder C.J., Tsimikas S., Witztum J.L. (2013). Adaptive immunity in atherogenesis: New insights and therapeutic approaches. J. Clin. Investig..

[6] Ridker P.M., Danielson E., Fonseca F.A., Genest J., Gotto A.M., Kastelein J.J., Koenig W., Libby P., Lorenzatti A.J., MacFadyen J.G. (2008). Rosuvastatin to prevent vascular events in men and women with elevated C-reactive protein. N. Engl. J. Med..

[7] Han K.H., Ryu J., Hong K.H., Ko J., Pak Y.K., Kim J.-B., Park S.W., Kim J.J. (2005). HMG-CoA Reductase Inhibition Reduces Monocyte CC Chemokine Receptor 2 Expression and Monocyte Chemoattractant Protein-1–Mediated Monocyte Recruitment In Vivo. Circulation.

[8] Lin H.-L., Ueng K.-C., Hsieh Y.-S., Chiang W.-L., Yang S.-F., Chu S.-C. (2012). Impact of MCP-1 and CCR-2 gene polymorphisms on coronary artery disease susceptibility. Mol. Biol. Rep..

[9] Martinovic I., Abegunewardene N., Seul M., Vosseler M., Horstick G., Buerke M., Darius H., Lindemann S. (2005). Elevated monocyte chemoattractant protein-1 serum levels in patients at risk for coronary artery disease. Circ. J. Off. J. Jpn. Circ. Soc..

[10] Nelken N.A., Coughlin S.R., Gordon D., Wilcox J.N. (1991). Monocyte chemoattractant protein-1 in human atheromatous plaques. J. Clin. Investig..

[11] Eriksson E.E. (2004). Mechanisms of leukocyte recruitment to atherosclerotic lesions: Future prospects. Curr. Opin. Lipidol..

[12] Hartmann C., Radermacher P., Wepler M., Nußbaum B. (2017). Non-Hemodynamic Effects of Catecholamines. Shock Inj. Inflamm. Sepsis Lab. Clin. Approaches.

[13] Schömig A. (1990). Catecholamines in myocardial ischemia. Systemic and cardiac release. Circulation.

[14] Gu H., Tang C., Yang Y. (2012). Psychological stress, immune response, and atherosclerosis. Atherosclerosis.

[15] Petidis K., Douma S., Doumas M., Basagiannis I., Vogiatzis K., Zamboulis C. (2008). The interaction of vasoactive substances during exercise modulates platelet aggregation in hypertension and coronary artery disease. BMC Cardiovasc. Disord..

[16] Wessel J., Moratorio G., Rao F., Mahata M., Zhang L., Greene W., Rana B.K., Kennedy B.P., Khandrika S., Huang P. (2007). C-reactive protein, an “intermediate phenotype” for inflammation: Human twin studies reveal heritability, association with blood pressure and the metabolic syndrome, and the influence of common polymorphism at catecholaminergic/beta-adrenergic pathway loci. J. Hypertens..

[17] Seeman T., Gruenewald T., Karlamangla A., Sidney S., Liu K., McEwen B., Schwartz J. (2009). Modeling multisystem biological risk in young adults: The Coronary Artery Risk Development in Young Adults Study. Am. J. Hum. Biol..

[18] Ulleryd M.A., Prahl U., Börsbo J., Schmidt C., Nilsson S., Bergström G., Johansson M.E. (2017). The association between autonomic dysfunction, inflammation and atherosclerosis in men under investigation for carotid plaques. PLoS ONE.

[19] Pagano G., Talamanca A.A., Castello G., Cordero M.D., d’Ischia M., Gadaleta M.N., Pallardó F.V., Pertović S., Tiano L., Zatterale A. (2014). Oxidative stress and mitochondrial dysfunction across broad-ranging pathologies: Toward mitochondria-targeted clinical strategies. Oxid. Med. Cell Longev..

[20] Tonhajzerova I., Sekaninova N., Olexova L.B., Visnovcova Z. (2020). Novel Insight into Neuroimmune Regulatory Mechanisms and Biomarkers Linking Major Depression and Vascular Diseases: The Dilemma Continues. Int. J. Mol. Sci..

[21] Köhler C.A., Freitas T.H., Maes M., De Andrade N.Q., Liu C.S., Fernandes B.S., Stubbs B., Solmi M., Veronese N., Herrmann N. (2017). Peripheral cytokine and chemokine alterations in depression: A meta-analysis of 82 studies. Acta Psychiatr. Scand..

[22] Kopschina Feltes P., Doorduin J., Klein H.C., Juárez-Orozco L.E., Dierckx R.A., Moriguchi-Jeckel C.M., de Vries E.F. (2017). Anti-inflammatory treatment for major depressive disorder: Implications for patients with an elevated immune profile and non-responders to standard antidepressant therapy. J. Psychopharmacol..

[23] Valkanova V., Ebmeier K.P., Allan C.L. (2013). CRP, IL-6 and depression: A systematic review and meta-analysis of longitudinal studies. J. Affect Disord..

[24] Eswarappa M., Neylan T.C., Whooley M.A., Metzler T.J., Cohen B.E. (2019). Inflammation as a predictor of disease course in posttraumatic stress disorder and depression: A prospective analysis from the Mind Your Heart Study. Brain Behav. Immun..

[25] Liu J.J., Bin Wei Y., Strawbridge R., Bao Y., Chang S., Shi L., Que J., Gadad B.S., Trivedi M.H., Kelsoe J.R. (2019). Peripheral cytokine levels and response to antidepressant treatment in depression: A systematic review and meta-analysis. Mol. Psychiatry.

[26] Lindqvist D., Dhabhar F.S., James S.J., Hough C.M., Jain F.A., Bersani F.S., Reus V.I., Verhoeven J.E., Epel E.S., Mahan L. (2017). Oxidative stress, inflammation and treatment response in major depression. Psychoneuroendocrinology.

[27] Manikowska K., Mikołajczyk M., Mikołajczak P.Ł., Bobkiewicz-Kozłowska T. (2014). The influence of mianserin on TNF-α, IL-6 and IL-10 serum levels in rats under chronic mild stress. Pharmacol. Rep..

[28] An H., Han J.W., Jeong H.-G., Kim T.H., Lee J.J., Lee S.B., Park J.H., Kim K.W. (2020). Parasympathetic predominance is a risk factor for future depression: A prospective cohort study. J. Affect. Disord..

[29] Ngampramuan S., Tungtong P., Mukda S., Jariyavilas A., Sakulisariyaporn C. (2018). Evaluation of Autonomic Nervous System, Saliva Cortisol Levels, and Cognitive Function in Major Depressive Disorder Patients. Depress. Res. Treat.

[30] Linnoila M., Karoum F., Calil H.M., Kopin I.J., Potter W.Z. (1982). Alteration of Norepinephrine Metabolism with Desipramine and Zimelidine in Depressed Patients. Arch. Gen. Psychiatry.

[31] Roy A., Pickar D., Linnoila M., Potter W.Z. (1985). Plasma Norepinephrine Level in Affective Disorders: Relationship to Melancholia. Arch. Gen. Psychiatry.

[32] Gehi A., Mangano D., Pipkin S., Browner W.S., Whooley M.A. (2015). Depression and heart rate variability in patients with stable coronary heart disease: Findings from the Heart and Soul Study. Arch. Gen. Psychiatry.

[33] Gorman J.M., Sloan R.P. (2000). Heart rate variability in depressive and anxiety disorders. Am. Heart J..

[34] La Rovere M.T., Bigger J.T., Marcus F.I., Mortara A., Schwartz P.J. (1998). Baroreflex sensitivity and heart-rate variability in prediction of total cardiac mortality after myocardial infarction. ATRAMI (Autonomic Tone and Reflexes after Myocardial Infarction) Investigators. Lancet.

[35] Benarroch E.E. (2019). Autonomic nervous system and neuroimmune interactions: New insights and clinical implications. Neurology.

[36] Jiang M., Qin P., Yang X. (2014). Comorbidity between depression and asthma via immune-inflammatory pathways: A meta-analysis. J. Affect Disord..

[37] Sperner-Unterweger B., Kohl C., Fuchs D. (2014). Immune changes and neurotransmitters: Possible interactions in depression?. Prog. Neuropsychopharmacol. Biol. Psychiatry.

[38] Laux L., Glanzmann P., Schaffner C.D., Spielberger C.D. (1981). Das State-Trait-Angstinventar (Testmappe Mit Handanweisung, Fragebogen STAI-G Form X 1 Und Fragebogen STAI-G Form X 2).

[39] Gaab J. (2009). PASA—Primary Appraisal Secondary Appraisal. Verhaltenstherapie.

[40] Zerssen D.v., Petermann F. (2011). B-LR—Beschwerden-Liste—Revidierte Fassung.

[41] Kirschbaum C., Pirke K.-M., Hellhammer D.H. (1993). The ‘Trier Social Stress Test’—A tool for investigating psychobiological stress responses in a laboratory setting. Neuropsychobiology.

[42] Breier A., Albus M., Pickar D., Zahn T.P., Wolkowitz O.M., Paul S.M. (1987). Controllable and uncontrollable stress in humans: Alterations in mood and neuroendocrine and psychophysiological function. Am. J. Psychiatry.

[43] Lechin F., Van Der Dijs B., Benaim M. (1996). Stress versus depression. Prog. Neuro-Psychopharmacol. Biol. Psychiatry.

[44] Bucolo C., Leggio G.M., Drago F., Salomone S. (2019). Dopamine outside the brain: The eye, cardiovascular system and endocrine pancreas. Pharmacol. Ther..

[45] Carney R.M., E Freedland K., Veith R.C., E Cryer P., A Skala J., Lynch T., Jaffe A.S. (1999). Major depression, heart rate, and plasma norepinephrine in patients with coronary heart disease. Biol. Psychiatry.

[46] Otte C., Neylan T.C., Pipkin S.S., Browner W.S., Whooley M.A. (2005). Depressive symptoms and 24-hour urinary norepinephrine excretion levels in patients with coronary disease: Findings from the Heart and Soul Study. Am. J. Psychiatry.

[47] Grossman F., Potter W.Z. (1999). Catecholamines in depression: A cumulative study of urinary norepinephrine and its major metabolites in unipolar and bipolar depressed patients versus healthy volunteers at the NIMH. Psychiatry Res..

[48] Potter W.Z., Manji H.K. (1994). Catecholamines in depression: An update. Clin. Chem..

[49] Frankenstein L., Zugck C., Schellberg D., Nelles M., Froehlich H., Katus H., Remppis A. (2009). Prevalence and prognostic significance of adrenergic escape during chronic beta-blocker therapy in chronic heart failure. Eur. J. Heart Fail.

[50] Lindahl B., Baron T., Erlinge D., Hadziosmanovic N., Nordenskjöld A., Gard A., Jernberg T. (2017). Medical Therapy for Secondary Prevention and Long-Term Outcome in Patients with Myocardial Infarction With Nonobstructive Coronary Artery Disease. Circulation.

[51] Santana M.M., Rosmaninho-Salgado J., Cortez V., Pereira F.C., Kaster M.P., Aveleira C.A., Ferreira M., Álvaro A.R., Cavadas C. (2015). Impaired adrenal medullary function in a mouse model of depression induced by unpredictable chronic stress. Eur. Neuropsychopharmacol. J. Eur. Coll. Neuropsychopharmacol..

[52] Lechin F., Van Der Dijs B., Orozco B., Lechin M.E., Baez S., Lechin A.E., Rada I., Acosta E., Arocha L., Jiménez V. (1995). Plasma neurotransmitters, blood pressure, and heart rate during supine-resting, orthostasis, and moderate exercise conditions in major depressed patients. Biol. Psychiatry.

[53] Goldstein D.S., Holmes C. (2008). Neuronal Source of Plasma Dopamine. Clin. Chem..

[54] Goldstein D.S., Mezey E., Yamamoto T., Aneman A., Friberg P., Eisenhofer G. (1995). Is there a third peripheral catecholaminergic system? Endogenous dopamine as an autocrine/paracrine substance derived from plasma DOPA and inactivated by conjugation. Hypertens. Res. Off. J. Jpn. Soc. Hypertens..

[55] Kuchel O. (1999). Peripheral dopamine in hypertension and associated conditions. J. Hum. Hypertens..

[56] Choi M.R., Rukavina Mikusic N.L., Kouyoumdzian N.M., Kravetz M.C., Fernández B.E. (2014). Atrial natriuretic peptide and renal dopaminergic system: A positive friendly relationship?. BioMed. Res. Int..

[57] Tomaschitz A., Ritz E., Kienreich K., Pieske B., März W., Boehm B.O., Drechsler C., Meinitzer A., Pilz S. (2012). Circulating dopamine and C-peptide levels in fasting nondiabetic hypertensive patients: The Graz Endocrine Causes of Hypertension study. Diabetes Care.

[58] Kox M., van Eijk L.T., Zwaag J., van den Wildenberg J., Sweep F.C.G.J., van der Hoeven J.G., Pickkers P. (2014). Voluntary activation of the sympathetic nervous system and attenuation of the innate immune response in humans. Proc. Natl. Acad. Sci. USA.

[59] van der Poll T., Coyle S.M., Barbosa K., Braxton C.C., Lowry S.F. (1996). Epinephrine inhibits tumor necrosis factor-alpha and potentiates interleukin 10 production during human endotoxemia. J. Clin. Investig..

[60] Köhler C.A., Freitas T.H., Stubbs B., Maes M., Solmi M., Veronese N., De Andrade N.Q., Morris G., Fernandes B.S., Brunoni A.R. (2018). Peripheral Alterations in Cytokine and Chemokine Levels after Antidepressant Drug Treatment for Major Depressive Disorder: Systematic Review and Meta-Analysis. Mol. Neurobiol..

[61] Hoogeveen R.C., Morrison A., Boerwinkle E., Miles J.S., Rhodes C.E., Sharrett A.R., Ballantyne C.M. (2005). Plasma MCP-1 level and risk for peripheral arterial disease and incident coronary heart disease: Atherosclerosis Risk in Communities study. Atherosclerosis.

[62] Di Battista A.P., Rhind S.G., Hutchison M.G., Hassan S., Shiu M.Y., Inaba K., Topolovec-Vranic J., Neto A.C., Rizoli S.B., Baker A.J. (2016). Inflammatory cytokine and chemokine profiles are associated with patient outcome and the hyperadrenergic state following acute brain injury. J. Neuroinflamm..

[63] Armaiz-Pena G.N., Gonzalez-Villasana V., Nagaraja A.S., Rodriguez-Aguayo C., Sadaoui N.C., Stone R.L., Matsuo K., Dalton H.J., Previs R.A., Jennings N.B. (2014). Adrenergic regulation of monocyte chemotactic protein 1 leads to enhanced macrophage recruitment and ovarian carcinoma growth. Oncotarget.

[64] Amirshahrokhi K., Khalili A.-R. (2016). Carvedilol attenuates paraquat-induced lung injury by inhibition of proinflammatory cytokines, chemokine MCP-1, NF-κB activation and oxidative stress mediators. Cytokine.

[65] Kudo H., Kai H., Kajimoto H., Koga M., Takayama N., Mori T., Ikeda A., Yasuoka S., Anegawa T., Mifune H. (2009). Exaggerated blood pressure variability superimposed on hypertension aggravates cardiac remodeling in rats via angiotensin II system-mediated chronic inflammation. Hypertension.

[66] Raedler T.J. (2011). Inflammatory mechanisms in major depressive disorder. Curr. Opin. Psychiatry.

[67] Kalkman H.O. (2019). Novel Treatment Targets Based on Insights in the Etiology of Depression: Role of IL-6 Trans-Signaling and Stress-Induced Elevation of Glutamate and ATP. Pharmaceuticals.

[68] von Dawans B., Ditzen B., Trueg A., Fischbacher U., Heinrichs M. (2019). Effects of acute stress on social behavior in women. Psychoneuroendocrinology.

[69] Wekenborg M.K., Von Dawans B., Hill L.K., Thayer J.F., Penz M., Kirschbaum C. (2019). Examining reactivity patterns in burnout and other indicators of chronic stress. Psychoneuroendocrinology.

[70] Jung F.U., Bae Y.J., Kratzsch J., Riedel-Heller S.G., Luck-Sikorski C. (2020). Internalized weight bias and cortisol reactivity to social stress. Cogn. Affect. Behav. Neurosci..

[71] Meier M., Wirz L., Dickinson P., Pruessner J.C. (2020). Laughter yoga reduces the cortisol response to acute stress in healthy individuals. Stress.

[72] Zhang Q., Ma J., Nater U.M. (2019). How Cortisol Reactivity Influences Prosocial Decision-Making: The Moderating Role of Sex and Empathic Concern. Front. Hum. Neurosci..

[73] Gecaite J., Burkauskas J., Brozaitiene J., Mickuviene N. (2019). Cardiovascular Reactivity to Acute Mental Stress: The importance of typed personality, trait anxiety, and depression symptoms in patients after acute coronary syndromes. J. Cardiopulm. Rehabil. Prev..

